# Heterogeneity in preferences for outcomes of integrated care for persons with multiple chronic diseases: a latent class analysis of a discrete choice experiment

**DOI:** 10.1007/s11136-022-03147-6

**Published:** 2022-05-18

**Authors:** Maaike Hoedemakers, Milad Karimi, Marcel Jonker, Apostolos Tsiachristas, Maureen Rutten-van Mölken

**Affiliations:** 1 Erasmus School of Health Policy & Management, Erasmus University Rotterdam, Rotterdam, The Netherlands; 2 Erasmus Choice Modelling Centre, Erasmus University Rotterdam, Rotterdam, The Netherlands; 3 Health Economics Research Centre, Nuffield Department of Population Health, University of Oxford, Oxford, UK; 4 Institute for Medical Technology Assessment, Erasmus University Rotterdam, Rotterdam, The Netherlands

**Keywords:** Integrated care, Multi-morbidity, Discrete choice experiment, Latent class analysis, Outcomes

## Abstract

**Purpose:**

For an integrated care programme to be successful, preferences of the stakeholders involved should be aligned. The aim of this study is to investigate to which extent outcomes beyond health are valued and to study the heterogeneity of preferences of those involved in integrated care.

**Methods:**

A discrete choice experiment (DCE) was conducted to elicit preferences for eight Triple Aim outcomes, i.e., physical functioning, psychological well-being, social relationships & participation, enjoyment of life, resilience, person-centeredness, continuity of care and total health and social care costs. Stakeholders were recruited among Dutch persons with multi-morbidity, informal caregivers, professionals, payers, and policymakers. A Bayesian mixed-logit model was used to analyse the data. Subsequently, a latent class analysis was performed to identify stakeholders with similar preferences.

**Results:**

739 stakeholders completed the DCE. Enjoyment of life was perceived as the most important outcome (relative importance: 0.221) across stakeholders, while total health and social care costs were perceived as least important (0.063). The latent class analysis identified four classes. The first class (19.9%) put most weight on experience with care outcomes. The second class (39%) favoured enjoyment of life. The third class (18%) focused relatively more on physical health. The fourth class (24%) had the least consistent preferences.

**Conclusion:**

This study has highlighted the heterogeneity in views of stakeholders in integrated care on what is important in health(care) for persons with multi-morbidity. To accurately value integrated care a variety of outcomes beyond health–e.g., enjoyment of life and experience with care–should be taken into account.

**Supplementary Information:**

The online version contains supplementary material available at 10.1007/s11136-022-03147-6.

## Introduction

Integrated care programmes that focus on multi-morbidity often include a package of complex and multifaceted interventions that have multiple aims [1, 2]. Such aims include improved population health, better patient experience, cost reduction (known as the Triple Aim [3]), and better experience of providing care (known as the Quadruple Aim [4]). Therefore, evaluations of such models of care require the measurement of a broad spectrum of outcomes that go beyond traditional health outcomes like health-related quality of life and longevity [5].

However, not all aims are expected to have the same importance to the different stakeholders involved in the design, provision, financing, and receipt of integrated care for persons with multi-morbidity. For example, patients may assign higher importance to experience with care than clinicians with overburdened workloads, while payers may be more sensitive to costs than other stakeholders. Discordance in preferences complicates the decision-making process [6]. International experience and scientific evidence show that the success of integrated care models is highly dependent on the alignment of stakeholder preferences for the model’s aims and achievements [7–9]. Hence, it is important to elicit their preferences and take them into account when designing and assessing integrated care for multi-morbidity [9].

A common technique to measure preferences in healthcare delivery is a discrete choice experiment (DCE), in which respondents are asked to make a number of choices between two hypothetical options characterised by attributes with differing levels [10]. A DCE forces respondents to make trade-offs between multiple elements or aims of a health care intervention [11]. Especially in integrated care this is important as interventions focus on improving outcomes beyond the quality-adjusted life year (QALY). An advantage of a DCE is that, by making patient-preferences so explicit, it makes it possible to incorporate them in decision-making [12]. To study heterogeneity in choices, one can use a latent class analysis to identify underlying subgroups of respondents with similar preferences and characterised by background characteristics. This information can be used to better understand differences in preferences between stakeholders and further align them.

The aim of this study was (1) to investigate to which extent outcomes beyond health are valued and (2) to study the heterogeneity of preferences for outcome measures of integrated care among stakeholders involved in integrated care. The outcome measures included in the preference study were physical functioning, psychological well-being, social relationships & participation, enjoyment of life, resilience, person-centeredness, continuity of care, and total health and social care costs. Respondents were recruited among persons with multi-morbidity, partners & other informal caregivers, professionals, payers, and policymakers. This is the first DCE study including such a wide variety of outcomes measures relevant to integrated care and such a diversity of stakeholders involved in integrated care.

## Methods

### Context of the DCE

This study took place in the context of the EU-funded SELFIE2020 project, in which we aimed to elicit preferences for outcome measures of integrated care that could be used in Multi-Criteria Decision Analysis [13], see box 1. In the current study, preference data from Dutch stakeholders involved in integrated care were used.

**Box 1:**
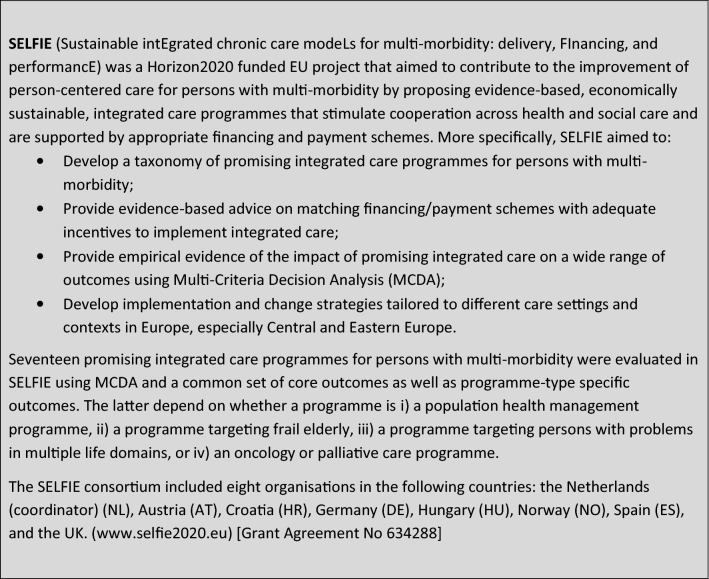
Information on the SELFIE project

### Attributes and levels

The development of attributes and attribute levels (see Table 1) consisted of two steps. First, a longlist with potentially relevant attributes was composed using four methods: (1) a literature review of outcome measures used in (integrated) care, (2) national workshops with patients, informal caregivers, professionals, payers and policymakers in the eight countries in the SELFIE project to discuss outcomes of integrated care, (3) eight focus groups with individuals with multi-morbidity to discuss what outcomes of integrated care matter to them [14], and (4) a review of outcomes being used in the 17 integrated care programmes in Europe that were evaluated in the SELFIE project. The second step was to shorten the list, a process that was guided by multiple criteria, including relevance to multi-morbidity in different contexts and population groups, non-redundancy, operationality, and preference independence [13]. The levels to describe the attributes were defined such that they represent the full range of the scale from worst to best, with an intermediate level in between. The wording of the levels was based on validated questionnaires that are used to measure these outcomes in empirical research [13]. For costs we used estimates of the mean total health and social care costs for people with multimorbidity in the Netherlands (middle level), which we increased and decreased by 20%.

**Table 1:** Attributes and levels

Attributes (outcome measures)	Levels
Physical functioning	1. *Severely* limited in physical functioning and activities of daily living
	2. *Moderately* limited in physical functioning and activities of daily living
	3. *Hardly or not at all* limited in physical functioning and activities of daily living
Psychological well-being	1. *Always or mostly* stressed, worried, listless, anxious, and down
	2. *Regularly* stressed, worried, listless, anxious, and down
	3. *Seldom or never* stressed, worried, listless, anxious, and down
Social relationships and participation	1. *No or barely any* meaningful connections with others
	2. *Some* meaningful connections with others
	3. *A lot* of meaningful connections with others
Enjoyment of life	1. *No or barely any* pleasure and happiness in life
	2. *Some* pleasure and happiness in life
	3. *A lot* of pleasure and happiness in life
Resilience	1. *Poor* ability to recover, adjust, and restore balance
	2. *Fair* ability to recover, adjust, and restore balance
	3. *Good* ability to recover, adjust, and restore balance
Person-centeredness	1. *Not or barely* person-centered
	2. *Somewhat* person-centered
	3. *Highly* person-centered
Continuity of care	1. *Poor* collaboration, transitions, and timeliness
	2. *Fair* collaboration, transitions, and timeliness
	3. *Good* collaboration, transitions, and timeliness
Total health- and social care costs	1. €8500 per participant per year
	2. €7000 per participant per year
	3. €5500 per participant per year

### Design

Given the large number of attributes, in combination with the three possible levels for each, a full factorial design that includes all 6561 possible alternatives (i.e. 3^8^: 8 attributes with 3 attribute levels), would not be feasible. To reduce this set of combinations to a manageable number, we used specialised software to select the most informative combinations of attribute levels per choice question, using Bayesian design algorithms that maximise the D-efficiency for a pre-specified conditional logit main-effects model [15–17]. Maximizing the D-efficiency involves minimizing the confidence sphere around the complete set of model parameters in this logit model. Priors for the weights of the attribute-levels, as required for an efficient optimisation approach, were obtained from literature [18]. To further improve the efficiency of the parameter estimates obtained from the DCE, the overall DCE design comprised ten different sub-designs. This means that instead of using 1 design for all respondents, we constructed 10 different sub-designs, and each respondent is only asked to complete one, randomly chosen, sub-design that consists of a pre-specified number of 18 choice tasks [15]. The informative priors from the literature were updated using the answers of the first 50 respondents from each stakeholder group, to create a more efficient DCE design for the remaining respondents in the stakeholder group.

When scanning the subsets of the full-choice design to find a D-optimal design we imposed two design constraints to reduce the complexity and to avoid unrealistic choice tasks. First, the highest level of enjoyment of life and lowest level of psychological well-being, and vice versa, were never combined within a single choice option, i.e., ‘Seldom or never stressed, worried, listless, anxious, and down’ (highest level of psychological well-being), and ‘No or barely any pleasure and happiness in life’ (lowest level of enjoyment of life) could never be part of the same programme description. Second, in each choice task either three or four attributes needed to have the same level for each alternative to reduce overall complexity and improve response efficiency [19].

### Questionnaire

**Fig. 1:**
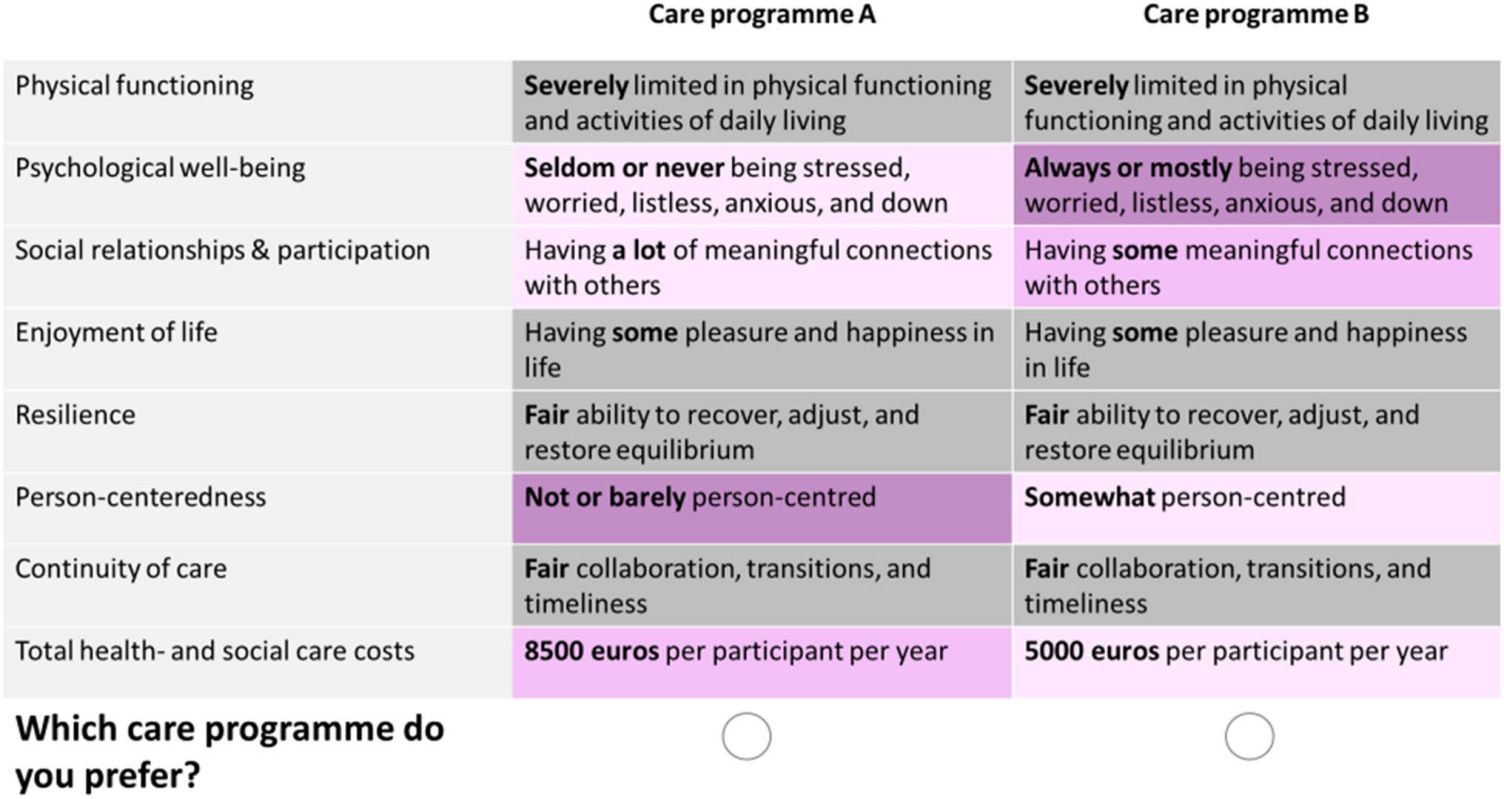
Example choice task DCE

The DCE questionnaire was self-administered and web-based. After informed consent, the meaning of the attributes and levels was explained. Each choice task described two alternatives with eight attributes of varying levels. These two alternatives represented two integrated care programmes with different outcome-profiles. These were labelled ‘Care programme A’ and ‘Care programme B’ (See Fig. 1 for an example choice task). Respondents were asked to complete two ‘warm-up’ DCE choice-tasks before the main choice tasks. This familiarised respondents with the attributes and levels and prepared them for the full set of 18 choice tasks. The choice tasks were presented in three groups of six choice tasks each, with a few general demographic or health-related questions in between to reduce the repetitive nature of the choice tasks. The questionnaire concluded with debriefing questions related to the stakeholder perspectives the respondents identify themselves with and the ease of understanding and completion of the choice tasks.

The DCE questionnaire was pilot tested with six patients, including think aloud sessions to see if there were elements that needed clarification. After the pilot study small changes were made in the visual design of the study to enhance the clarity of the questionnaire.

### Subject recruitment and data collection

To recruit a representative group of different stakeholders involved in integrated care, we aimed to recruit 750 respondents among persons with multi-morbidity (*n* = 150), informal caregivers of persons with multi-morbidity (*n* = 150), professionals (*n* = 150), payers (*n* = 150) and policymakers (*n* = 150). In July 2017, members of an online marketing research panel who were persons with multi-morbidity, informal caregivers, or professional care providers were invited to complete the questionnaire. Payers and policymakers were invited via the same panel organisation, but since it was difficult to reach 150 respondents in these groups, recruitment was supplemented by personal invitations of payers and policymakers in the network of the researchers of the SELFIE project, followed by snowballing. Between July 2017 and July 2018, we approached healthcare payers such as health insurance companies and departments of municipalities responsible for paying social care. To include policymakers, we invited public servants working at the ministry of health, provincial or local governments, official governmental advisory bodies, mayors, aldermen, and city councillors with health and/or social care in their portfolio. Each participant was asked to confirm that they belonged to their assigned stakeholder group and to respond from that perspective. They were also invited to indicate one or more other stakeholder group(s) that they felt they belonged to as well. It was made impossible to fill in the questionnaire using a mobile phone or tablet as the choice task would not be fully visible.

### Statistical analysis

First, a Bayesian mixed logit model (MIXL), using diffuse priors for the mean values of the random coefficients, was used to analyse the data of all respondents simultaneously. This model allowed all utility coefficients to be randomly distributed and estimated a full covariance matrix among them. A burn-in phase of 10,000 Markov Chain Monte Carlo (MCMC) draws was used, followed by 30,000 draws to reliably approximate the posterior. The relative importance of each attribute (i.e., outcome measure of integrated care) was based on the coefficient of its best level (level 3) divided by the sum of all best attribute levels.

Second, a latent class model was used to model heterogeneity across individuals with a discrete distribution over a set of classes, and individuals were sorted into a set of classes based on their observed choice data [20]. Latent class analysis is an extension of the standard logit model and is used to identify unobserved groups of similar individuals (latent classes) with homogeneous preferences based on observed variables. These distinct groups can possess (widely) different preferences regarding integrated care. Furthermore, latent class modelling is probabilistic, which means that respondents are allocated to the group they are most likely to be a member of. Subsequently, posterior analysis can be used to describe differences in characteristics across groups. Initially, we compared 2 to 9 class solutions for the best statistical fit based on the Bayesian Information Criterion (BIC) and Consistent Aikake Information Criterion (CAIC), with a lower value implying a better fit [21]. We also considered theoretical interpretability and the size of the classes to see if another number of classes would be more logical based on the observed variables. For each respondent, the posterior probability that (s)he belongs to each latent class was calculated and each respondent was assigned to the class with the highest probability. Class membership was based solely on estimated preferences from the DCE. To assess whether differences between classes were significant, we conducted chi-square tests (categorical variables), one-way ANOVA tests (continuous variables) and Kruskal Wallis tests (non-parametric test for continuous variables).

Third, as the observed preference heterogeneity could be related to stakeholders having multiple roles, we investigated which other perspectives the stakeholders identified themselves with, in a descriptive analysis.

Analyses were performed in Stata 16.0, using the bayesmixedlogit module specified with Metropolis-within-Gibbs sampling and default (uninformative) priors for the MIXL model and the lclogit procedure for the latent class models.

## Results

### Study population

There were 935 persons that started the questionnaire and gave informed consent, of which 739 (79%) finished all DCE scenarios. The mean time to complete the questionnaire was approximately 20 min. 705 respondents spent at least 5 min filling in the questionnaire and all further analyses are performed on this group.

Table 2 summarizes the characteristics of the respondents. Their mean age was 49.6 years, 54.2% was female, and the majority (73.1%) of respondents was employed. The respondents’ highest attained educational level was relatively high. 31% of the respondents reported no health problems.

**Table 2:** Respondent characteristics

Participation	
Started questionnaire and gave informed consent *n*	935
Finished all DCE scenario’s *n*	739
Mean time to completion (SD)	19.90 (12.53)
5–90 min *n*	705
Demographics (*N* = 705)	
Mean age (SD)	49.59 (14.05)
Median age (min–max)	51 (21–88)
Gender – female *n* (%)	382 (54)
Educational level *n* (%)	
1. Low	25 (4)
2. Medium	188 (27)
3. High	492 (70)
Work status *n* (%)	
1. Paid job	515 (73)
2. Volunteer work	136 (19)
3. Retired / pre-pension	103 (15)
4. [Partially] Work disabled	44 (6)
5. Looking for a job	19 (3)
6. Do not have paid job	12 (2)
7. Housewife/househusband	63 (9)
8. Student	36 (5)
Health characteristics	
General health *n* (%)	
1. Excellent	99 (14)
2. Very good	166 (24)
3. Good	282 (40)
4. Fair	133 (19)
5. Poor	25 (4)
Mean general health (SD)	2.74 (1.03)
Health conditions (top 10 most frequent) *n* (%)	
1. Depression, anxiety or emotional difficulties	86 (12)
2. Colon problem, irritable bowel or colitis	82 (12)
3. Chronic back pain or sciatica	67 (10)
4. Diabetes	66 (9)
5. Osteoarthritis (not rheumatoid arthritis)	60 (9)
6. Asthma	42 (6)
7. Rheumatoid arthritis	42 (6)
8. Chronic bronchitis, COPD or emphysema	41 (6)
9. Heart disease, angina, heart attack, bypass surgery or angioplasty	40 (6)
10. Stomach problem, ulcer, gastritis or reflux	38 (5)
No health problems	217 (31)
Other health problems	102 (14)
I prefer not to answer	27 (4)
Mean number of health problems (SD)	1.79 (2.08)
Stakeholder group *n* (%)	
1. Person with multi-morbidity	158 (22)
2. Informal caregiver	152 (22)
3. Professional	148 (21)
4. Payer	102 (14)
5. Policymaker	145 (21)
Difficulty DCE choice tasks *n* (%)	
1. Very easy	25 (4)
2. Easy	181 (26)
3. Not too easy, not too difficult	291 (42)
4. Difficult	175 (25)
5. Very difficult	18 (3)
Mean difficulty (SD)	2.97 (0.87)

### DCE preferences across all stakeholders

**Fig. 2:**
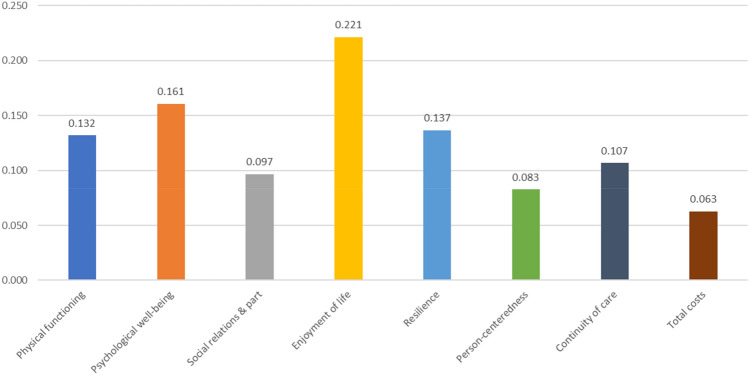
Relative importance of the outcome measures. (Note: All relative importance weights sum up to 1. The relative importance of each outcome measure was based on the coefficient of its attribute-level 3 divided by the sum of all level 3 coefficients. For instance, ‘Enjoyment of life’ had a coefficient of 5.571 for its level-3 attribute (see Table 3), and it yielded a relative importance weight of 5.571/25.164 = 0.221, where 25.164 was the sum of level-3 coefficients across all domains.)

The results of the Bayesian MIXL (Table 3) showed that all attribute-levels differed from level 1. The attribute levels had the expected positive sign and the coefficients of level 3 were always larger than those of level 2. This means that level 2 and level 3 were valued higher than level 1 and the level 3 was valued higher than level 2. On average, the respondents assigned the highest relative importance to enjoyment of life, followed by psychological well-being, and resilience (Fig. 2). The least important outcome was total health and social care costs. However, the standard deviations of all attribute (levels) indicated a wide variation in preferences among respondents (Table 3).

**Table 3:** Attribute-level coefficients of the Bayesian MIXL model

Attribute (i.e., outcome measure)	Level	Mean	95% Credible interval	Standard deviation	95% Credible interval
Physical functioning	2	2.29	2.03–2.55	2.13	1.86–2.41
	3	3.32	2.97–3.67	3.13	2.78–3.51
Psychological well-being	2	2.02	1.79–2.25	1.79	1.55–2.04
	3	4.04	3.65–4.44	3.54	3.16–3.95
Social relationships & participation	2	1.74	1.53–1.95	1.54	1.33–1.77
	3	2.43	2.17–2.69	2.19	1.92–2.47
Enjoyment of life	2	3.61	3.30–3.92	2.30	2.03–2.60
	3	5.57	5.11–6.04	3.75	3.35–4.16
Resilience	2	2.54	2.31–2.77	1.61	1.39–1.86
	3	3.44	3.14–3.74	2.19	1.92–2.50
Person-centeredness	2	1.26	1.08–1.45	1.27	1.06–1.47
	3	2.09	1.85–2.32	1.91	1.67–2.17
Continuity of care	2	2.05	1.83–2.27	1.67	1.43–1.92
	3	2.69	2.43–2.95	2.12	1.83–2.43
Total health- and social care costs	2	0.74	0.59–0.90	1.13	0.96–1.33
	3	1.58	1.37–1.80	1.87	1.63–2.13

The coefficients represent the respondent’s preferences for the various attributes and their levels. Each attribute consisted of 3 levels, with level 3 as best performing level. A higher coefficient reflects a higher preference

### Grouping stakeholders with similar preferences

**Fig. 3:**
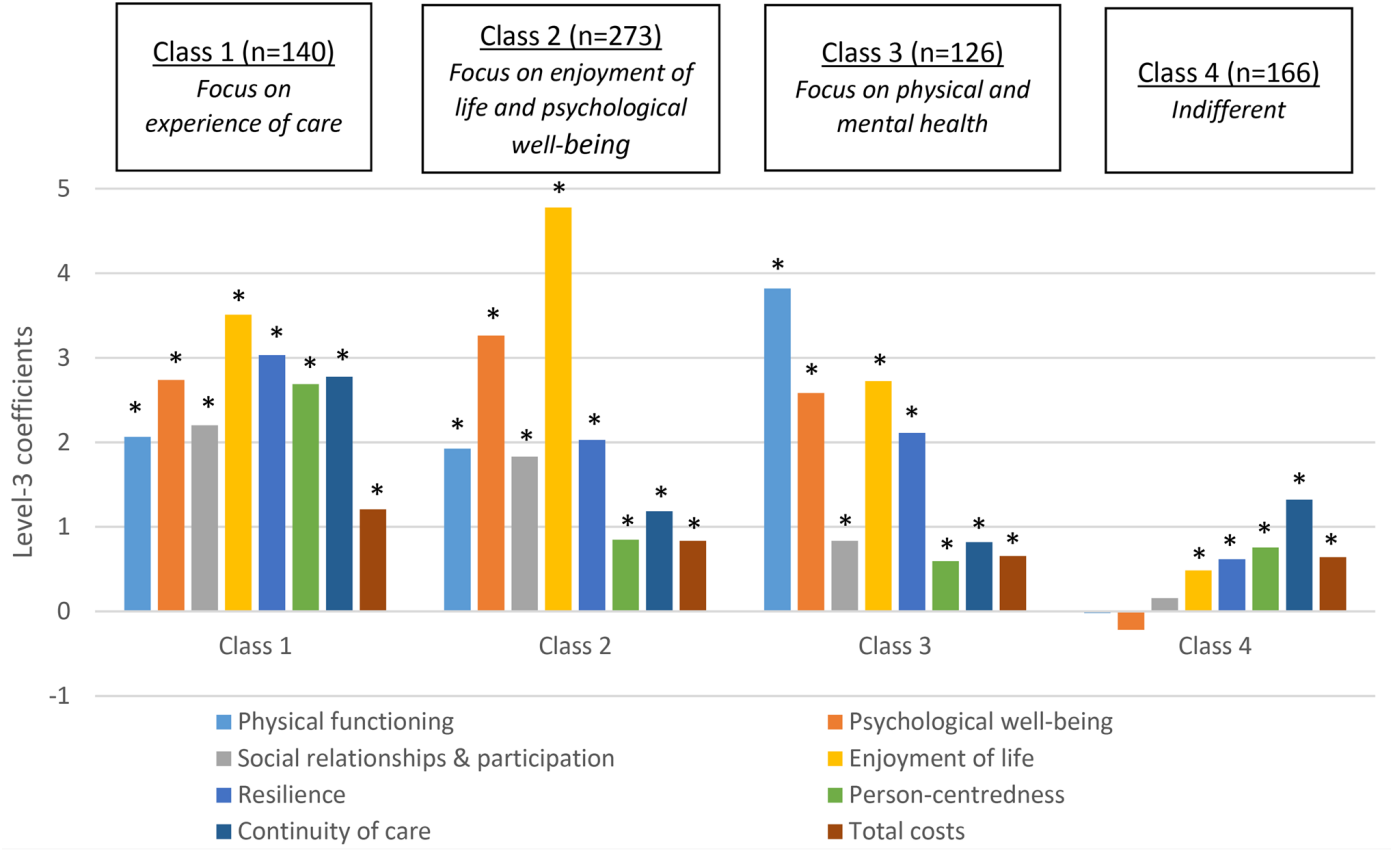
Results latent class analysis: coefficients of attribute-level 3. *Significant preference within the class (*P* < 0.05)

Based on the information criteria (BIC and CAIC), the latent class model with four classes provided the best model fit. Based on the class probabilities 20% (*n* = 140) of all respondents was assigned to class 1, 39% (*n* = 273) to class 2, 18% (*n* = 126) to class 3, and 24% (*n* = 166) to class 4. The average of the respondents’ maximum posterior class membership probabilities was 0.82 (SD = 0.17, median = 0.87), varying from 0.74 for class 1 and 0.89 for class 4. Figure 3 presents the class-specific preference coefficients. The estimates in class 1, 2 and 3 had the expected direction, i.e., respondents preferred a higher level of each outcome. In class 4 the preferences for physical functioning, psychological well-being and social relationships & participation were not statistically significant. A table with all coefficients, including standard errors and p-values, is presented in online Appendix 1.

Compared to the other classes, class 1 respondents had the highest estimates for the experience with care outcomes (continuity of care and person-centeredness). In class 2, respondents assigned a relatively higher weight to enjoyment of life, followed by psychological well-being, than the other classes. Respondents in class 3 had a stronger preference for physical health than the other classes, followed by three outcomes related to mental health i.e., psychological well-being, enjoyment of life and resilience. The coefficients in class 4 were overall quite small, which indicates that the preferences were less consistent than in the other classes.

### Characteristics of stakeholders with similar preferences

Table 4 presents the background characteristics of stakeholders in the four latent classes. All classes included representatives from all primary stakeholder groups, although professionals were overrepresented in class 1 (26%), followed by policymakers (25%). Class 1 had the highest share of females (62%). Also, health was best in this class compared to the other classes based on both self-perceived general health (mean 2.54 and 44% of respondents who answered “excellent” or “very good”) and the mean number of health conditions (1.55).

**Table 4:** Class-specific respondent characteristics

	Class 1	Class 2	Class 3	Class 4	*P*-value
	*n* = 140	*n* = 273	*n* = 126	*n* = 166	
Participation					
Time to completion–mean (SD)	20.36 (13.61)	20.77 (13.02)	19.84 (11.80)	18.16 (11.17)	0.000
Time to completion–median (min–max)	17.31 (6.97–85.07)	17.35 (5.13–85.07)	16.58 (5.75–77.95)	15.74 (5.13–83.38)	
Demographics– *n* (%)					
Age–mean (SD)	48.59 (14)	47.63 (14)	49.56 (14)	53.67 (14)	0.000
Gender (female)	87 (62)	156 (57)	52 (41)	87 (52)	0.000
Educational level					0.000
1. Low	5 (4)	9 (3)	2 (2)	9 (5)	
2. Medium	32 (23)	59 (22)	33 (26)	64 (39)	
3. High	103 (74)	205 (75)	91 (72)	93 (56)	
Work status					0.000
1. Paid job	102 (73)	216 (79)	99 (79)	98 (59)	
2. Volunteer work	30 (21)	53 (19)	16 (13)	37 (22)	
3. Retired / pre-pension	23 (16)	30 (11)	15 (12)	35 (21)	
4. [Partially] Work disabled	8 (6)	12 (4)	10 (8)	14 (8)	
5. Looking for a job	3 (2)	7 (3)	3 (2)	6 (4)	
6. Do not have paid job	5 (4)	4 (1)	2 (2)	1 (1)	
7. Housewife/househusband	11 (8)	25 (9)	6 (5)	21 (13)	
8. Student	7 (5)	17 (6)	5 (4)	7 (4)	
Health characteristics					
General health *n* (%)					0.000
1. Excellent	24 (17)	39 (14)	20 (16)	16 (10)	
2. Very good	38 (27)	68 (25)	30 (24)	30 (18)	
3. Good	58 (41)	113 (41)	43 (34)	68 (41)	
4. Fair	18 (13)	46 (17)	25 (20)	44 (27)	
5. Poor	2 (1)	7 (3)	8 (6)	8 (5)	
Mean general health (SD)	2.54 (1)	2.69 (1)	2.77 (1)	2.99 (1)	0.000
Health conditions* n* (%)					
1. Colon problem, irritable bowel or colitis	15 (11)	36 (13)	15 (12)	16 (10)	
2. Depression, anxiety or emotional difficulties	10 (7)	32 (12)	16 (13)	28 (17)	
3. Chronic back pain or sciatica	14 (10)	20 (7)	14 (11)	19 (11)	
4.Osteoarthritis (not rheumatoid arthritis)	14 (10)	20 (7)	7 (6)	19 (11)	
5. Chronic bronchitis, COPD or emphysema	7 (5)	17 (6)	7 (6)	10 (6)	
6. Diabetes	5 (4)	16 (6)	15 (12)	30 (18)	
7. Heart disease, angina (chest pain from heart problem), heart attack, bypass surgery or angioplasty	8 (6)	16 (6)	9 (7)	7 (4)	
8. Rheumatoid arthritis	8 (6)	15 (5)	6 (5)	13 (8)	
9. Stomach problem, ulcer, gastritis or reflux	9 (6)	15 (5)	2 (2)	12 (7)	
10. Asthma	6 (4)	14 (5)	11 (9)	11 (7)	
11. Poor circulation in your legs	8 (6)	14 (5)	8 (6)	9 (5)	
12. Thyroid disorder	6 (4)	14 (5)	6 (5)	7 (4)	
13. Cancer during the past five years	3 (2)	5 (2)	5 (4)	11 (7)	
14. Congestive heart failure	3 (2)	2 (1)	4 (3)	9 (5)	
No health problems	49 (35)	93 (34)	38 (30)	37 (22)	
Other health problems	20 (14)	41 (15)	12 (10)	29 (17)	
I prefer not to answer	4 (3)	10 (4)	3 (2)	10 (6)	
Multi-morbidity *n* (%)					
1 health problem	29 (21)	53 (20)	23 (19)	35 (22)	
2 health problems	16 (12)	38 (14)	17 (14)	17 (11)	
3 or more health problems	32 (24)	67 (25)	43 (35)	57 (37)	
Mean number of health problems (SD)	1.55 (2.21)	1.62 (1.88)	1.85 (1.84)	2.23 (2.41)	0.000
Median number of health problems (min–max)	1 (0–15)	1 (0–7)	1 (0–9)	1 (0–11)	
Stakeholder group *n* (%)					
Stakeholder group					0.000
1. Person with multi-morbidity	27 (19)	57 (21)	36 (29)	38 (23)	
2. Informal caregiver	24 (17)	51 (19)	21 (17)	56 (34)	
3. Professional	37 (26)	62 (23)	22 (17)	27 (16)	
4. Payer	17 (12)	50 (18)	18 (14)	17 (10)	
5. Policymaker	35 (25)	53 (19)	29 (23)	28 (17)	
Number of additional perspectives chosen by respondent *n* (%)					
0 additional perspectives	64 (47)	139 (51)	63 (50)	68 (43)	
1 additional perspective	51 (37)	101 (37)	46 (37)	68 (43)	
2 or more additional perspectives	22 (16)	30 (11)	16 (13)	22 (14)	
Mean number of additional perspectives chosen (SD)	0.74 (0.0.84)	0.63 (0.76)	0.65 (0.77)	0.73 (0.77)	0.000
Additional perspective *n* (%)*					
Additional perspective					0.000
1. Person with multi-morbidity	21 (15)	37 (14)	17 (13)	28 (17)	
2. Informal caregiver	30 (22)	45 (17)	20 (16)	33 (21)	
3. Professional	16 (12)	26 (10)	14 (11)	16 (10)	
4. Payer	14 (10)	20 (7)	15 (12)	21 (13)	
5. Policymaker	20 (15)	41 (15)	15 (12)	18 (11)	
Difficulty* n* (%)					
Difficulty					0.000
1. Very easy	1 (1)	10 (4)	4 (3)	10 (6)	
2. Easy	35 (26)	71 (26)	34 (27)	41 (26)	
3. Not too easy, not too difficult	62 (45)	113 (42)	57 (46)	59 (37)	
4. Difficult	37 (27)	71 (26)	25 (20)	42 (27)	
5. Very difficult	2 (1)	5 (2)	5 (4)	6 (4)	

*This percentage is based on the number of respondents that chose a certain perspective divided by the total number of respondents in the respective class, i.e., the numbers do not vertically add up to the *n* of the class, as some respondents chose no additional perspective

In class 2 the stakeholder groups were quite evenly distributed, with somewhat more professionals (23%). This class consisted of respondents with the lowest mean age (47.6 years). Furthermore, the educational level and employment rate were the highest in this class. 75% of the respondents had a high educational level and 79% currently had a paid job.

Persons with multi-morbidity were more frequently a member of class 3 (29%). This class, which predominantly consisted of males (59%), also had a worse health status than respondents in class 1 and 2.

Class 4 had the highest share of informal caregivers (34%). The respondents in this class were on average older (53.7 years), lower educated, in worse general health (31% “fair” or “poor” health) and had more health problems (mean 2.23 health problems) than respondents in the other classes. Of the respondents in this class, 30% found the questionnaire difficult or very difficult, although the time to completion was the fastest of all classes (18.2 min).

### Multiple perspectives per stakeholder

Of the respondents, 48% chose no additional stakeholder perspective they identify themselves with. 39% of the respondents only selected one additional perspective, 13% identified with two or more additional perspectives. In the entire sample, 18% of the persons with multi-morbidity identified themselves as informal caregiver (Table 5). Of the informal caregivers, 29% identified themselves as person with multi-morbidity. 30% of the professionals identified themselves with the perspective of informal caregiver. 59% of the payers viewed themselves as policymaker. Of the policymakers, 24% selected informal caregiver as additional perspective.

**Table 5:**
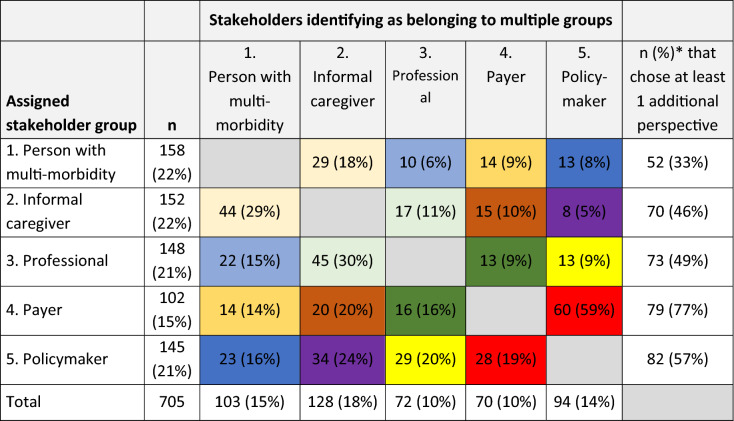
Overlap in perspectives of respondents

^*^The numbers do not add up to this total because respondents were allowed to select more than one additional stakeholder perspective. The color scheme indicates the following: for example, the purple category consists of policymakers that also identify as informal caregiver, and of informal caregivers that also identify as policymaker

When comparing the four classes (Table 4), class 4 had the lowest percentage of respondents that chose no additional perspective (43%). In all four classes ‘Informal caregiver’ was the most frequent additional perspective and in classes 1, 3 and 4 this was followed by person with multi-morbidity. There were no marked differences between the classes in the additional perspectives that were chosen.

## Discussion

### Interpretation of the main findings

This study investigated stakeholders’ preferences for outcomes of integrated care for persons with multi-morbidity using a DCE. Based on the mixed logit model results of the pooled data, which showed that all attribute levels were statistically different from 1, it was established that all outcome measures and all levels of the outcomes measures influenced stakeholders’ choices. This means that stakeholders took all outcome measures into account when deciding upon which care programme was preferred. Enjoyment of life, psychological well-being and resilience were deemed most important and total health and social care costs least important, but there was a lot of variation in preferences.

When divided into four classes using latent class analysis, we could identify a class that assigned a relatively higher weight to the two experience with care outcomes, i.e., continuity of care and person-centeredness, a class that emphasized the importance of enjoyment of life and psychological well-being, a class that was more focused on physical functioning and a class with inconsistent preferences. Each of the classes included persons with multi-morbidity as well as informal caregivers, professionals, payers and policy makers, suggesting that differences between the classes were not particularly driven by stakeholder group. One of the reasons that differences in preferences cannot be directly related to stakeholder perspective might be that respondents obviously have multiple roles, as was clearly shown by their self-reported additional stakeholder perspectives. Many informal caregivers, payers and policy makers were patients themselves.

Nevertheless, some stakeholders were overrepresented in some classes. The class that focused more on experience with care (class 1) included relatively more professionals and policy makers, i.e., stakeholders that are used to monitoring process outcomes as indicators of the quality of care. In the Dutch context, where patients are free to choose between care providers, professionals are incentivized to improve patient’s experience and satisfaction with their services to increase their market share. This is emphasized by payers who consider quality indicators when contracting providers. The class that focused on physical and mental health (class 3) had the highest share of persons with multi-morbidity, resulting in the highest share of persons reported having more than one health problem. A likely explanation is that people with immediate concerns about their health prefer outcomes related to these domains in contrast to experience with care or cost outcomes. In the class with less consistent preferences (class 4), persons with multi-morbidity and informal caregivers were overrepresented. Respondents in this class found the questionnaire difficult, which has likely contributed to the inconsistency. The fact that they had the shortest completion time might illustrate this difficulty.

The largest class (class 2) with approximately 39% of the sample, consisted of respondents that put much weight on enjoyment of life. The respondents were quite evenly distributed over the stakeholders. The respondents in this class were relatively younger, higher educated, healthier or more likely to have a paid job than respondents in class 3 and 4. Their lesser experience with (physical) health problems might explain their higher valuation of enjoyment of life.

### Comparison with other research

In contrast to our study, most previous DCE-studies include the perspective of one stakeholder group, e.g., patients or healthcare workers, or compare the preferences of two stakeholder groups [22, 23]. Furthermore, many health-related DCE-research include attributes related to characteristics of the new therapies or drugs (i.e., structure-attributes such as waiting time till appointment, care provider/setting or process-attributes such as shared decision making) [24] whereas in the current study we included outcomes of the intervention.

In a previous paper, covering preference data from 8 European countries (including these Dutch data) [18], we also compared different stakeholder groups directly and reported considerable within-country agreement between stakeholders involved in integrated care with enjoyment of life ranking first and costs ranking last. However, we also found that patients assigned significantly higher values to physical functioning than professionals in five countries, which is in line with our finding that class 3, which focused more on physical health, contained the highest proportion of persons with multi-morbidity.

Similar to our study, other studies acknowledge the importance of measuring a broader set of outcomes than merely the physical and mental health outcomes that are traditionally included in health-related quality of life [25, 26]. This is required to fully capture the outcomes that interventions are trying to achieve. The discussion on outcomes beyond the quality-adjusted life year (QALY) largely concentrates on interventions in the care sector, such as elderly care or care for physically or mentally disabled people. In that context, a lot of attention is being paid to well-being outcomes, for which several questionnaires were developed in recent years [27]. Well known instruments include the Adult Social Care Outcomes Toolkit (ASCOT) [28] and the ICEpop CAPability measure for Older people (ICECAP- O) [29, 30]. The ASCOT focuses on social care related quality of life and, similar to our outcome measures, also includes ‘social participation’ as one of their 8 domains. The ICECAP-O is conceptually based on the capability approach and one of the five domains covered in this instrument is ‘enjoyment’ which received the highest weight in our study. A more recent instrument is the Well-being Of Older People measure [31] that captures relevant well-being domains for older people–among which multi-morbidity is common–and includes e.g., ‘resilience and acceptance’ and ‘social contacts’. These outcomes were also included in the current DCE, in which resilience was in the top 3 outcomes that received the highest importance. Another example is the extension of the EQ-5D into the EuroQOL Health and Well-being (EQ-HWB) [32], which also includes outcomes in social care and carers’ quality of life. Similarly, in our study we included social relationships and participation.

### Strengths and limitations

This study is one of the first that elicited weights for a set of outcomes that goes beyond health, and requires trade-offs between health, well-being, experience, and costs to obtain weights. It included a sufficiently large representation of multiple stakeholder groups involved in integrated care for multi-morbidity. Furthermore, the inclusion of a variety of background characteristics and information on self-perceived health, allowed us to investigate the differences between stakeholders that had different opinions on the importance of the outcome measures. What is unique for this study is that we also asked the stakeholders for other roles they might have. We have learned that the additional perspective(s) that were chosen did not explain the variation in preferences between the classes.

Several limitations in the current study should also be mentioned. The survey could only be completed using a computer and not via a mobile phone. Therefore, younger persons may be underrepresented. Secondly, although the sample is quite large, payers are less well represented among the stakeholder groups. Moreover, although a DCE is a widely used method to elicit preferences in health care and health care delivery, it also has its limitations. One of the main concerns regarding DCEs is the external validity due to hypothetical bias, i.e., the disparity between stated preferences based on hypothetical DCE questions and revealed preferences based on actual choices in real life [33, 34]. Recently, a number of case studies reported a high external validity of DCEs, with over 90% of the individual choices correctly predicted, thus suggesting a high degree of confidence [35]. However, this research does not pertain to integrated care, nor does it use outcome measures as attributes.

For future research, it would be interesting to further investigate the reasons behind differences in preferences. Preferences are, for example, likely to be influenced by a person’s own experiences, or the experiences of significant others [36, 37]. In the current study we did not explicitly ask about this.

### Implications

Information about these preferences can be used in the design of new integrated care initiatives by concentrating on interventions that specifically aim to improve well-being and by better targeting interventions to patients’ preferences. As this study has shown that patients’ preferences cannot be presumed based on their characteristics, obtaining more insight in an individual’s preferences should be an important part of a shared decision-making process.

The preferences can also be used in health care evaluation. Currently, health care evaluation focuses mainly on health status, life expectancy and QALYs, although there is a demand for tools that incorporate multiple outcomes that emerge from interventions with benefits beyond health [38, 39]. The current study showed that outcomes related to well-being, and mental health in particular, were highly valued. More specifically, enjoyment of life received much weight in both the full sample analysis, and 3 out of 4 latent classes in which it received the highest or second highest weight. Yet it is not a common outcome measure in health care research. Future evaluations of integrated care interventions that measure a similar set of outcomes can make use of the weights obtained in this study. That enables the calculation of weighted outcomes which can be combined in an overall value score using multi-criteria decision analysis (MCDA) [11].

## Conclusion

Stakeholders involved in integrated care for multi-morbidity value the outcome measure ‘enjoyment of life’ most and ‘total health and social care costs’ least. There is considerable heterogeneity in preferences, with a group of stakeholders assigning relatively higher importance to experience with care outcomes, a group assigning relatively higher importance to enjoyment of life and psychological wellbeing and a group focusing more on physical health. Differences in preferences were only weakly related to whether respondents were patients, informal caregivers, professionals, payers or policymakers as many stakeholders have multiple roles. This heterogeneity in preferences underlines the need to measure a wide range of different outcome measures when evaluating integrated care, including well-being outcomes and experience with care outcomes.

## Supplementary Information

Below is the link to the electronic supplementary material.

Supplementary file1 (DOCX 21 kb)

## Data Availability

Due to the nature of this research, participants of this study did not agree for their data to be shared publicly, so supporting data is not available.

## Code availability

The code that supports the findings of this study is available upon request.
