# Supplementary material for: Heterogeneity in preferences for outcomes of integrated care for persons with multiple chronic diseases: a latent class analysis of a discrete choice experiment
Source: Qual Life Res. 2022 May 18;31(9):2775–89. doi: 10.1007/s11136-022-03147-6 (PMC9356934; doi:10.1007/s11136-022-03147-6)
Supplement: Supplementary file 1 — Supplementary file1 (DOCX 21 kb) [file 11136_2022_3147_MOESM1_ESM.docx]

**Appendix**

**Appendix 1.** Latent class model

| **Outcome** | **Coefficient** | **Std. Err.** | **P-value** | **[95% Conf. Interval]** | |
| --- | --- | --- | --- | --- | --- |
| **Class 1** | | | | | |
| Physical functioning level 2 | 1.201 | 0.288 | 0.000 | 0.636 | 1.766 |
| Physical functioning level 3 | 2.065 | 0.435 | 0.000 | 1.213 | 2.918 |
| Psychological well-being level 2 | 1.229 | 0.261 | 0.000 | 0.718 | 1.740 |
| Psychological well-being level 3 | 2.737 | 0.423 | 0.000 | 1.909 | 3.566 |
| Social relationships level 2 | 1.430 | 0.204 | 0.000 | 1.030 | 1.829 |
| Social relationships level 3 | 2.202 | 0.281 | 0.000 | 1.652 | 2.753 |
| Enjoyment of life level 2 | 2.165 | 0.252 | 0.000 | 1.672 | 2.659 |
| Enjoyment of life level 3 | 3.511 | 0.410 | 0.000 | 2.706 | 4.315 |
| Resilience level 2 | 2.165 | 0.252 | 0.000 | 1.670 | 2.660 |
| Resilience level 3 | 3.031 | 0.311 | 0.000 | 2.422 | 3.641 |
| Person-centeredness level 2 | 1.462 | 0.203 | 0.000 | 1.065 | 1.859 |
| Person-centeredness level 3 | 2.689 | 0.289 | 0.000 | 2.123 | 3.255 |
| Continuity of care level 2 | 2.149 | 0.237 | 0.000 | 1.684 | 2.613 |
| Continuity of care level 3 | 2.777 | 0.275 | 0.000 | 2.237 | 3.317 |
| Total costs level 2 | 0.394 | 0.160 | 0.014 | 0.080 | 0.709 |
| Total costs level 3 | 1.208 | 0.222 | 0.000 | 0.773 | 1.643 |
| **Class 2** | | | | | |
| Physical functioning level 2 | 1.380 | 0.129 | 0.000 | 1.126 | 1.633 |
| Physical functioning level 3 | 1.926 | 0.174 | 0.000 | 1.584 | 2.268 |
| Psychological well-being level 2 | 1.600 | 0.131 | 0.000 | 1.343 | 1.858 |
| Psychological well-being level 3 | 3.264 | 0.190 | 0.000 | 2.892 | 3.636 |
| Social relationships level 2 | 1.224 | 0.108 | 0.000 | 1.012 | 1.436 |
| Social relationships level 3 | 1.830 | 0.128 | 0.000 | 1.578 | 2.081 |
| Enjoyment of life level 2 | 3.061 | 0.192 | 0.000 | 2.684 | 3.438 |
| Enjoyment of life level 3 | 4.778 | 0.267 | 0.000 | 4.254 | 5.301 |
| Resilience level 2 | 1.480 | 0.118 | 0.000 | 1.248 | 1.712 |
| Resilience level 3 | 2.028 | 0.149 | 0.000 | 1.737 | 2.319 |
| Person-centeredness level 2 | 0.575 | 0.106 | 0.000 | 0.368 | 0.783 |
| Person-centeredness level 3 | 0.848 | 0.128 | 0.000 | 0.597 | 1.099 |
| Continuity of care level 2 | 0.961 | 0.112 | 0.000 | 0.741 | 1.180 |
| Continuity of care level 3 | 1.185 | 0.130 | 0.000 | 0.931 | 1.439 |
| Total costs level 2 | 0.469 | 0.091 | 0.000 | 0.290 | 0.649 |
| Total costs level 3 | 0.835 | 0.119 | 0.000 | 0.602 | 1.069 |
| **Class 3** | | | | | |
| Physical functioning level 2 | 2.571 | 0.225 | 0.000 | 2.130 | 3.012 |
| Physical functioning level 3 | 3.819 | 0.316 | 0.000 | 3.199 | 4.440 |
| Psychological well-being level 2 | 1.157 | 0.169 | 0.000 | 0.826 | 1.488 |
| Psychological well-being level 3 | 2.583 | 0.249 | 0.000 | 2.095 | 3.070 |
| Social relationships level 2 | 0.915 | 0.146 | 0.000 | 0.629 | 1.202 |
| Social relationships level 3 | 0.836 | 0.189 | 0.000 | 0.465 | 1.207 |
| Enjoyment of life level 2 | 1.757 | 0.188 | 0.000 | 1.389 | 2.126 |
| Enjoyment of life level 3 | 2.722 | 0.264 | 0.000 | 2.204 | 3.240 |
| Resilience level 2 | 1.552 | 0.184 | 0.000 | 1.192 | 1.912 |
| Resilience level 3 | 2.110 | 0.227 | 0.000 | 1.665 | 2.556 |
| Person-centeredness level 2 | 0.378 | 0.138 | 0.006 | 0.107 | 0.649 |
| Person-centeredness level 3 | 0.595 | 0.175 | 0.001 | 0.251 | 0.939 |
| Continuity of care level 2 | 0.558 | 0.156 | 0.000 | 0.251 | 0.864 |
| Continuity of care level 3 | 0.819 | 0.174 | 0.000 | 0.478 | 1.160 |
| Total costs level 2 | 0.332 | 0.139 | 0.017 | 0.059 | 0.605 |
| Total costs level 3 | 0.654 | 0.177 | 0.000 | 0.306 | 1.001 |
| **Class 4** | | | | | |
| Physical functioning level 2 | 0.150 | 0.102 | 0.139 | -0.049 | 0.350 |
| Physical functioning level 3 | -0.022 | 0.119 | 0.856 | -0.256 | 0.212 |
| Psychological well-being level 2 | 0.142 | 0.093 | 0.127 | -0.041 | 0.325 |
| Psychological well-being level 3 | -0.218 | 0.143 | 0.128 | -0.499 | 0.063 |
| Social relationships level 2 | 0.146 | 0.095 | 0.122 | -0.039 | 0.332 |
| Social relationships level 3 | 0.155 | 0.115 | 0.177 | -0.070 | 0.380 |
| Enjoyment of life level 2 | 0.504 | 0.105 | 0.000 | 0.298 | 0.711 |
| Enjoyment of life level 3 | 0.485 | 0.140 | 0.001 | 0.210 | 0.760 |
| Resilience level 2 | 0.558 | 0.098 | 0.000 | 0.366 | 0.750 |
| Resilience level 3 | 0.615 | 0.117 | 0.000 | 0.385 | 0.845 |
| Person-centeredness level 2 | 0.484 | 0.092 | 0.000 | 0.303 | 0.665 |
| Person-centeredness level 3 | 0.756 | 0.123 | 0.000 | 0.515 | 0.997 |
| Continuity of care level 2 | 0.936 | 0.114 | 0.000 | 0.713 | 1.158 |
| Continuity of care level 3 | 1.321 | 0.129 | 0.000 | 1.068 | 1.573 |
| Total costs level 2 | 0.318 | 0.087 | 0.000 | 0.148 | 0.487 |
| Total costs level 3 | 0.641 | 0.102 | 0.000 | 0.441 | 0.842 |
|  |  |  |  |  |  |
| share1 |  |  |  |  |  |
| _cons | -0.225 | 0.220 | 0.307 | -0.657 | 0.207 |
|  |  |  |  |  |  |
| share2 |  |  |  |  |  |
| _cons | 0.428 | 0.183 | 0.019 | 0.070 | 0.787 |
|  |  |  |  |  |  |
| share3 |  |  |  |  |  |
| _cons | -0.257 | 0.214 | 0.230 | -0.677 | 0.163 |
